# A Prospective Evaluation of Missed Injuries in Trauma Patients, Before and After Formalising the Trauma Tertiary Survey

**DOI:** 10.1007/s00268-013-2226-z

**Published:** 2013-10-01

**Authors:** Gerben B. Keijzers, Don Campbell, Jeffrey Hooper, Nerolie Bost, Julia Crilly, Michael Craig Steele, Chris Del Mar, Leo M. G. Geeraedts

**Affiliations:** 1 Emergency Department, Gold Coast Health Service District, Gold Coast University Hospital, 1 Hospital Boulevard, Southport, QLD 4215 Australia; 2 School of Medicine, Bond University, Gold Coast, QLD Australia; 3 Statewide Emergency Department Clinical Network, Gold Coast University Hospital, 1 Hospital Boulevard, Southport, QLD 4215 Australia; 4 Research Centre for Clinical and Community Practice Innovation, Griffith University, PO Box 7057, Gold Coast, QLD 4222 Australia; 5 Faculty of Business, Bond University, Gold Coast, QLD Australia; 6 Public Health, School of Medicine, Bond University, Gold Coast, QLD Australia; 7 Department of Surgery, VU University Medical Centre, PO Box 7057, 1007 MB Amsterdam, The Netherlands; 8 School of Medicine, Griffith University, Gold Coast, QLD Australia; 9 Griffith Graduate Research School, Griffith University, Gold Coast, QLD Australia

## Abstract

**Objective:**

This study prospectively evaluated in-hospital and postdischarge missed injury rates in admitted trauma patients, before and after the formalisation of a trauma tertiary survey (TTS) procedure.

**Methods:**

Prospective before-and-after cohort study. TTS were formalised in a single regional level II trauma hospital in November 2009. All multitrauma patients admitted between March–October 2009 (preformalisation of TTS) and December 2009–September 2010 (post-) were assessed for missed injury, classified into three types: Type I, in-hospital, (injury missed at initial assessment, detected within 24 h); Type II, in-hospital (detected in hospital after 24 h, missed at initial assessment and by TTS); Type III, postdischarge (detected after hospital discharge). Secondary outcome measures included TTS performance rates and functional outcomes at 1 and 6 months.

**Results:**

A total of 487 trauma patients were included (pre-: *n* = 235; post-: *n* = 252). In-hospital missed injury rate (Types I and II combined) was similar for both groups (3.8 vs. 4.8 %, *P* = 0.61), as were postdischarge missed injury rates (Type III) at 1 month (13.7 vs. 11.5 %, *P* = 0.43), and 6 months (3.8 vs. 3.3 %, *P* = 0.84) after discharge. TTS performance was substantially higher in the post-group (27 vs. 42 %, *P* < 0.001). Functional outcomes for both cohorts were similar at 1 and 6 months follow-up.

**Conclusions:**

This is the first study to evaluate missed injury rates after hospital discharge and demonstrated cumulative missed injury rates >15 %. Some of these injuries were clinically relevant. Although TTS performance was significantly improved by formalising the process (from 27 to 42 %), this did not decrease missed injury rates.

## Introduction

A common quality indicator in trauma care is *missed injury* [1, 2]. Missed injuries are the result of the prioritisation that takes place during the initial assessment and management in the emergency department (ED) and emergency intervention. Because the focus in the ED is on making time-critical decisions, complete injury identification during resuscitation (including primary and secondary survey) is not always feasible [3–5].

Performance of a trauma tertiary survey (TTS) within 24 h has been suggested as a tool to address this problem and minimise the risk of missed injuries [3]. The TTS should follow the episode of emergency care (primary and secondary survey and emergency interventions). It comprises a comprehensive general physical reexamination and review of all investigations, including diagnostic imaging and blood results, within 24 h [4–6] and again when the patient is conscious, cooperative, and mobilised [3, 6, 7].

The TTS would be expected to reduce missed injuries and therefore improve trauma care. However, our recent systematic review [8] found the evidence to support this is suboptimal. Among the deficiencies was the substantial variation in outcome definitions for *missed injury* (leading to a recommendation for a classification focused on consistent outcome definitions, as outlined in Box 1). None reported missed injury rates after hospital discharge (Type III) nor functional (long-term) outcomes [3, 5, 7, 9–13].

**Box 1:** Missed injury classification [8]

Missed injury type	Description
I	Before TTS, or as result of TTS—in-hospital
	Injury missed at initial assessment (primary and secondary survey and emergency intervention), but detected within 24 h, before or through formal TTS (delayed diagnosis at 24 h). (i.e., injury missed at initial assessment)
II	After TTS, in-hospital
	Injury missed by TTS, detected in hospital after 24 h (i.e., injury missed at initial assessment *and* TTS)
III	After TTS, after hospital discharge
	Injury missed during hospital stay including TTS, detected after hospital discharge (i.e., injury missed at initial assessment *and* TTS *and* hospital stay)

*TTS* tertiary trauma survey

A retrospective study [14] in our facility found poor compliance to routine TTS and identified a lack of data regarding postdischarge missed injuries. As a result, we pragmatically evaluated prospectively the missed injury rates during hospital stay (Types I and II) as well as after discharge (Type III) in trauma patients in our level II facility, before and after implementation of a formalised TTS procedure.

## Materials and methods

### Design and setting

A prospective cohort study with before-and-after design was conducted on trauma patients who were admitted to the Gold Coast Hospital between March 2009 and October 2010. The Gold Coast Hospital is a public teaching hospital and is the designated, level II [15], regional trauma hospital for the area and covers all major specialties, excluding cardiac surgery and burns. The ED had 67,000 presentations in 2010, and the hospital had no dedicated trauma service or formalised process for review of admitted trauma patients. Patients were managed at the discretion of the admitting consultant and team. The Human Research Ethics Committees of the Gold Coast Health Service District and Bond University approved the study.

### Patients

All admitted multitrauma patients were identified prospectively. Patients eligible for study inclusion were aged 16 years and older and admitted for at least 24 h, AND met any of the four following criteria: (1) injuries in two or more body regions, (2) a high impact mechanism (high-speed motor vehicle collision, pedestrian versus car, fall from >1.5 meter), (3) chest or abdominal injuries, or (4) diagnosed with a fractured neck of femur younger than aged 65 years. These inclusion criteria were based on previous work [14]. Patients were identified using the Emergency Department Information System (EDIS) and the hospital based corporate information system (HBCIS). The resultant database was complemented with data from the Queensland Trauma Registry (QTR). All patients or their proxies were asked to provide written consent for a 1- and 6-month telephone follow-up interview.

## Implementation of practice change (formalised TTS—intervention)

During a 3-week period in November 2009, a hospital-wide practice change was implemented via a formalised TTS procedure. Implementation involved (i) the provision of TTS forms (Appendix 1) to trauma admitting wards, (ii) repeated education for all levels of medical and nursing staff working on these wards on the use of the TTS form, and (iii) a directive from the surgical departmental head for TTS form completion as part of routine care within 24 h of admission.

Before the implementation of the formalised TTS (preintervention period, March 2009 to 3 November 2009) routine care was provided at the discretion of the admitting team. The treating team performed TTS at their discretion, based on their clinical judgment and without standardised forms. Trauma admitting teams provided the same care following the practice change (postintervention period, 28 November 2009 to September 2010) except for the use the formalised and standardised TTS form. Data collection procedures were identical in both time periods.

### Data collection

We prospectively identified eligible patients and reviewed their medical records. A previously used data collection tool (Appendix 2) was used to assess the documentation of the relevant admission [15]. Data collected by the trained research nurse included demographic variables, mechanism of injury, Australasian Triage Scale category (ATS) [16], and Glasgow Coma Scale (GCS) on arrival. If no GCS was documented, but the patient was noted to be “alert,” this was coded as a GCS of 15. The QTR provided the Injury Severity Score (ISS) [17] scores for our dataset. An ISS score of greater than 15 indicated severe trauma.

Data related to the inpatient admission included whether a TTS was documented during admission and which components of the TTS were performed. A scripted follow-up telephone interview (Appendix 3) was conducted at 1 and 6 months after discharge. This follow-up interview collected data on missed injuries after discharge, complications of care, return to preinjury function, and ongoing medical care requirements. If not contactable during initial phone call, up to five attempts were made by the research nurse at varying times and days for both the 1- and the 6-month follow-up interviews.

If patients indicated during follow-up that an injury was missed during their hospital stay, this prompted review of relevant medical records and imaging reports by a consultant emergency physician, who determined whether this was a true missed injury. An injury was only classified as “missed injury” if there was no documentation (in either medical record or radiology report) of the reported injury during the hospital stay. Musculoskeletal injuries included fractures and lacerations where soft-tissue injuries were defined as other musculoskeletal injuries, such as contusions, grazes and haematomas that caused pain, swelling, or lack of function. The emergency physician also could classify the reported “injury” as a complication of the injury (i.e., paresthesia, chronic pain) or complication of care (such as postoperative infection or venous thromboembolism). Patients who reported a missed injury were offered appropriate pathways for follow-up. Finally, a search of the Death Registry (Queensland Registry for Births, Deaths and Marriages) was undertaken to identify the mortality rate at 6 months posthospital discharge and cause of death.

The primary outcome was missed injury rate. Three types of missed injuries have been defined previously [8] and are shown in Box 1. This study prospectively evaluated the in-hospital missed injury rate (Types I and II combined) and postdischarge missed injury rate (Type III) before and after the implementation of a formalised TTS. Secondary outcome measures included TTS performance rates and functional outcomes at 1 and 6 months posthospital discharge.

### Sample size and statistical analysis

We anticipated an overall missed injury rate (Types I, II, and III combined) of 15 % in the pre-period and expected the formalised TTS to reduce the missed injury rate to 5 %. Using an alpha of 0.05 and a power of 0.8, a sample size of 110 per group was required. Because we anticipated a telephone follow-up rate of 45 %, a total of 244 patients was required for both cohorts.

Before analysis, the accuracy of patient inclusion and demographic data were checked with the QTR database. Discordant data fields (<5 %) were reviewed and corrected. Deidentified data was analysed using SPSS v17.0 software (SPPS Inc., Chicago, IL). For continuous variables, we used an independent *t* test and analysis of variance (ANOVA) to compare demographic groups. For categorical variables, the Chi square test was used to compare differences in proportions. A *P* value ≤0.05 was deemed statistically significant.

This study is reported to adhere to the STROBE statement (www.strobe-statement.org).

## Results

The baseline characteristics of the pre- and post-practice change cohorts (*n* = 235 and *n* = 252, respectively) are summarised in Table 1. The cohorts are comparable in demographics, such as age, gender, and injury severity, although a higher percentage of patients in the post-cohort was admitted to the intensive care unit (ICU): 24 vs. 33 %, *P* = 0.02. Mortality was not significantly different (2.6 vs. 1.2 %, *P* = 0.26; Table 2).

**Table 1:** Characteristics of the study population

Characteristics	Preintervention, *n* = 235	Postintervention, *n* = 252
Age, year mean (SD)	40.4 (17)	41.1 (19)
Male, *n* (%)	169 (72)	194 (79)
ISS score, median (IQR)	9 (12)	10 (12)
ISS >15, *n* (%)	56 (24)	61 (26)
GCS <15, *n* (%)	54 (23)	50 (20)
Mechanism of Injury		
MVA, high speed, *n* (%)	25 (11)	15 (6)
MVA, moderate speed, *n* (%)	26 (11)	36 (14)
MBA, *n* (%)	41 (17)	47 (19)
Fall from height >1.5 metres, *n* (%)	49 (21)	62 (25)
Pedestrian vs. car, *n* (%)	16 (7)	11 (4)
Other blunt mechanism, *n* (%)	78 (33)	82 (32)
Disposition from ED		
Surgical ward, *n* (%),	88 (37)	89 (35)
Orthopedic ward, *n* (%)	77 (33)	76 (30)
ICU, *n* (%)	56 (24)	84 (33)^*^
Other, *n* (%)	13 (6)	4 (2)^*^

*SD* standard deviation, *IQR* interquartile range, *ISS* injury severity score, *GCS* Glasgow coma scale, *MVA* motor vehicle accident, *MBA* motor bike accident, *ED* emergency department, *ICU* intensive care unit

* *P* < 0.05

**Table 2:** Trauma tertiary survey and missed injuries

TTS elements and missed injuries	Preintervention, *n* = 235	Postintervention, *n* = 252
TTS performed, *n* (%)	65 (27)	106 (42)^***^
Major components of TTS, *n* (%)		
C-spine	26 (40)	60 (57)^*^
Chest	32 (49)	100 (94)^***^
Abdomen	49 (75)	100 (94)^***^
Pelvis	10 (15)	57 (54)^***^
Back	4 (6)	53 (50)^***^
Missed injuries		
Type I and II (combined)		
Detected in-hospital, *n*/*N* (%)	9/235 (3.8)	12/252 (4.8)
Type III		
Detected post hospital discharge		
At 1 month, *n*/*N* (%)	18/131 (13.7)	14/122 (11.5)
At 6 months, *n*/*N* (%)	4/105 (3.8)	3/92 (3.3)
Mortality, *n* (%)	6 (2.6)	3 (1.2)

*TTS* trauma tertiary survey, *n* number of events, *N* population at follow-up

* *P* < 0.05; ** *P* < 0.01; *** *P* < 0.001

### Missed injuries in-hospital (Types I and II combined)

The rate of combined Types I and II (in-hospital) missed injuries was similar for both cohorts (pre-: 3.8 % vs. post-: 4.8 %, *P* = 0.61; Tables 2, 3).

**Table 3:** Type and time of missed injuries and complications detected

Missed injury (type and time) and complication	Preintervention, *n*/*N* (%)	Postintervention, *n*/*N* (%)
Types I and II (combined)—detected in-hospital	9/235 patients (3.8 %)	12/252 patients (4.8 %)
	Total injuries = 11	Total injuries = 18
	(9 × C, 3 × R, 2 × I)	(15 × C, 5 × R, 3 × I)
	1× finger # (C)	2× lacerations (2 × I)
	1× facial # (R, I)	1× tibia plateau # (R, I)
	1× sternum # (C)	1× ankle # (R, C)
	1× calcaneum # (R, C)	3x abdominal pain (3xR, 3xC)
	1× hearing loss (R, C)	
	6× STI (5 × C, 1 × I)	11× STI (11 × C)
Type III—detected at 1 month after discharge	18/131 patients (13.7 %)	14/122 patients (11.5 %)
	(15 × C, 5 × R, 3 × I)	(12 × C, 2 × R, 2 × I)
	13 × STI (12 × C, 1 × I)	1× distal radius # (R, C)
	2× scaphoid # (2 × R, 2 × C)	1× L4 # (R, C)
	1× mandible # (R, I)	12 × STI (10 × C, 2 × I)
	1× dislocated toes (R, I)	
	1× laceration scalp (R, C)	
Type III—detected at 6 months after discharge	4/105 patients (3.7 %)	3/93 patients (3.3 %)
	(4 × C, 2 × R)	(2 × C, 3 × R, 1 × I)
	1× ulnar styloid # (R, C)	1× patella # (R, C)
	1× visual loss (R, C)	1× rib # (R, C)
	2× STI (2 × C)	1× tooth # (R, I)
Complications		
Reported at 1 month	11/131 (8.4 %)	10/122 (8.2 %)
	5× DVT	1× DVT
	1× PE/arrest	1× PE
	3× headache/dizzy	4× paresthesia
	2× infection	4× infection
Reported at 6 months	7/105 (6.7 %)	8/92 (8.7 %)
	1× DVT	1× DVT
	3× chronic pain	2× chronic pain
	1× vertigo	3× STI
	1× PTSD	1× paresthesia
	1× seizures	1× infection

*n* number of events, *N* total population, *#* fracture, *STI* soft-tissue injury, *DVT* deep vein thrombosis, PE pulmonary embolism, *PTSD* posttraumatic stress disorder, *C* conservatively managed, *R* referral to specialist, *I* surgical intervention required

### Missed injuries postdischarge (Type III)—at 1 and 6 months

Patients in the both pre- and post-cohorts reported similar rates of missed injuries during follow-up telephone interview at 1 month (13.7 vs. 11.5 %, *P* = 0.59) and 6 months (3.8 vs. 3.3 %, *P* = 0.84; Table 3). Complication rates also were similar for both cohorts at 1 and 6 months (Table 3). Most injuries were musculoskeletal or soft-tissue in nature. Of the soft-tissue injuries, four required intervention (ligamentous cervical spine injury required a neck brace, two knee injuries required surgery for cruciate ligament injury, and one shoulder injury required surgery for rotator cuff injury).

### TTS performance

The implementation of a formalised TTS on trauma admitting wards substantially improved TTS performance (pre- 27 % vs. post- 42 %, *P* < 0.001). All major components of reexamination were significantly more frequently performed in the post-cohort (Table 2).

### Missed injuries by TTS performance

Both in pre- and post-cohorts, more injuries were detected in-hospital (Types I and II) when a TTS was performed compared with when this was not done (pre- 6/65 = 9.2 % vs. 3/170 = 1.8 %, *P* = 0.008; post- 10/106 = 9.4 % vs. 2/145 = 1.4 %, *P* = 0.003).

There was no difference in Type III injury detection at 1 month and 6 months between patients who received a formal TTS compared with those who did not in either the pre- cohort (1 month 5/28 = 17.9 % vs. 13/103 = 12.6 %, *P* = 0.54; 6 months 2/23 = 8.7 % vs. 2/82 = 2.5 %, *P* = 0.21) or post-cohort (1 month 4/41 = 9.8 % vs. 10/81 = 12.3 %, *P* = 0.77; 6 months 1/28 = 3.6 % vs. 2/64 = 3.1 %, *P* = 1.00).

### Functional outcomes—at 1 and 6 months

The proportion of patients returning to almost normal or normal level of functioning was similar for both cohorts at 1 month (pre- 20 % vs. post- 17 %, *P* = 0.61) and 6 months (pre- 39 % vs. post- 44 %, *P* = 0.53; Tables 4, 5). Of the patients who returned to work or university, there was no difference in average hours worked at 1 month (pre- 31 h vs. post- 34 h, *P* = 0.29) and 6 months (pre- 39 h vs. post- 36 h, *P* = 0.22). Both cohorts had similar proportions of ongoing follow-up, mainly general practitioner (GP), physiotherapy, and occupational therapy.

**Table 4:** 1-month functional outcomes

1 month outcomes	Preintervention, *n* = 132	Postintervention, *n* = 122
Returned to prelevel functioning in		
ADL, *n* (%)		
Yes	8 (6)	4 (3)
Almost	18 (14)	17 (14)
Difficulty with some ADL	38 (29)	34 (28)
Difficulty with most ADL	64 (49)	64 (53)
Unable	3 (2)	3 (3)
Hours at work, mean h (SD)		
Preinjury, *n* = 106, *n* = 91	39 (15)	43 (11)
1 month post injury, *n* = 41, *n* = 28	31 (14)	34 (10)
Ongoing follow-up		
GP	85 (64)	71 (59)
Physiotherapist	40 (30)	36 (30)
OT	15 (11)	18 (15)
(Community) nurse	17 (13)	5 (4)^*^
Psychologist	3 (2)	3 (3)

*ADL* activities of daily living, *SD* standard deviation, *GP* general practitioner, *OT* occupational therapist

* *P* < 0.05

**Table 5:** 6-month functional outcomes

6-month outcomes	Preintervention, *n* = 105	Postintervention, *n* = 92
Returned to prelevel functioning in		
ADL, *n* (%)		
Yes	20 (19)	21 (23)
Almost	21 (20)	19 (21)
Difficulty with some ADL	36 (35)	29 (32)
Difficulty with most ADL	27 (26)	22 (25)
Unable	1 (1)	1 (1)
Hours at work, mean h (SD)		
Preinjury, *n* = 90, *n* = 74	40 (13)	41 (12)
6-month post injury, *n* = 64, *n* = 57	39 (14)	36 (13)
Ongoing follow-up		
GP	47 (45)	46 (50)
Physiotherapist	58 (55)	43 (47)
OT	13 (12)	10 (11)
(Community) nurse	10 (10)	7 (8)
Psychologist	6 (6)	7 (8)

*ADL* activities of daily living, *SD* standard deviation, *GP* general practitioner, *OT* occupational therapist

* *P* < 0.05

## Discussion

We did not find a difference in missed injury rates (in-hospital and after discharge) as a result of the implementation of a formalised TTS procedure. However, this study is the first to report on missed injuries after hospital discharge (Type III) related to TTS performance in the multitrauma population. We found a substantial cumulative missed injury rate 1 and 6 months after hospital discharge. Approximately 1 in 6 (between 15 and 18 %) of the patients who were available for follow-up reported Type III missed injuries, either at 1 or 6 months. Of these patients with Type III missed injuries (*n* = 39 in both cohorts combined), almost one-third (*n* = 12) required a specialist referral, and 13 % of these (*n* = 5) required surgical intervention. Furthermore, this is the first study that reports on functional outcomes and ongoing health care consumption related to the TTS. We did not find any differences in measured outcomes.

The in-hospital (Types I and II) missed injury rate (pre- 3.8 % vs. post- 4.8 %) was consistent with previous literature, including a retrospective review in the same hospital (in-hospital missed injury rate of 3.3 %) and a systematic review [8, 15] reporting an average in-hospital missed injury rate of 4.3 %.

The (Type III) postdischarge missed injury rate reported by patients at 1- and 6-month follow-up was three- to fourfold higher than our in-hospital missed injury rate. Potential explanations for this finding include, but are not limited to: (1) serial physical examination or TTS were not performed or incomplete; (2) abnormal physical examination or radiology findings were incorrectly interpreted (misdiagnosis); (3) abnormal findings were diagnosed, but not documented.

We did not find an overall effect of formalising the TTS, with similar missed injury rates and functional outcomes in both cohorts. Despite the significant increase in TTS performance, this practice improvement may not have been enough to have an effect on missed injury rates. Whilst we implemented measures to optimise TTS performance (e.g., involving key stakeholders and providing repeated education sessions), the compliance of the TTS was still not performed in more than half of the patients. This may be explained by the pragmatic, real-world nature of this study and possible reasons include: lack of (institutional) governance regarding trauma care, staff turnover, perceived loss of autonomy, high clinical workload, external pressures and difficulties in achieving a (cultural) change in behaviour—a problem not unique to our study [18–20].

Although we did not find a difference between the two cohorts, the analysis of missed injury rate by TTS performance suggests that any form of TTS, either routine (pre-) or formal (post-), increases in-hospital missed injury detection (Types I and II) but has little effect on injuries post postdischarge (Type III). Because this was not part of the original hypothesis and this comparison is potentially flawed due to possible selection bias, this data should be interpreted with caution.

## Limitations

The limited success of changing practice from routine non-standardised TTS to the formalised TTS forms procedure is likely due to the pragmatic nature of this study however there are some further limitations. First, this is a single-site study, which may limit the ability to generalise the results. However, the in-hospital missed injury rate is consistent with the existing literature, suggesting the practice in our institution is not likely to be markedly different from others. Second, as we were unable to identify a validated published instrument that measured the postdischarge outcomes of interest, we created our own scripted telephone interview. Functional outcomes we measured included: return to preinjury level of activities, hours worked, as well as the requirement to have further medical or allied health involvement after hospital discharge. Further validation of these post discharge outcomes in this population is recommended. Third, the follow-up rates at 1 (~50 %) and 6 months (~40 %) may have introduced selection bias. Although our telephone response rates compare favourably to other studies [20, 21], patients who were unable to be interviewed may have had similar or more missed injuries. In the unlikely situation that none of the patients lost to follow up had a missed injury, the overall postdischarge (Type III) missed injury rate would still be considerable at 8 % (39/487). Fourth, we relied on self-report of new injuries during the telephone interviews, but this is unlikely to have lead to systematic error between the two cohorts. We were unable to determine the accuracy of self-reporting of complications such as chronic pain and DVT. Furthermore, there was a large proportion of patients with musculoskeletal and soft-tissue injuries. This is consistent with the literature [8]. Although soft-tissue injury was the final diagnosis, clinical review and/or diagnostic imaging often was required. Although the majority of patients did not require intervention, some patients still had delayed recovery as a result. Lastly, although we accessed the Queensland Death registry, we cannot rule out that patients died in other states or countries.

## Conclusions

This is the first study to describe missed injury rates after hospital discharge in relation to TTS performance. However, attempting to improve tertiary survey rates by pragmatically formalising the process did not have a significant effect on (in-hospital—Types I and II, or postdischarge—Type III) missed injury rates. We attribute this to the real-world nature of this study with sub-optimal increase in TTS performance (from 27 to 42 %) as a result of this practice change.

The focus of the literature up to date has been on in-hospital missed injuries (Type I and Type II) and this is the first study report on post-discharge missed injuries (Type III), with several requiring consultant referral and surgical intervention. A simple checklist (such as a standardised TTS form) may not be enough, and a multifaceted, system approach may be required to address the problem of patients returning to the community with undiagnosed injuries. One solution may be improved governance, for example by implementation of a dedicated trauma service.
